# Aqua­(dicyanamido-κ*N* ^1^)(2,9-dimethyl-1,10-phenanthroline-κ^2^ *N*,*N*′)(nitrato-κ^2^ *O*,*O*′)cobalt(II)–2,9-dimethyl-1,10-phenanthroline–water (2/1/2)

**DOI:** 10.1107/S1600536811035148

**Published:** 2011-09-14

**Authors:** Feng-Hua Cui, Pei-Zheng Zhao

**Affiliations:** a College of Chemistry and Environmental Science, Henan Normal University, Xinxiang 453007, People’s Republic of China; b Department of Engineering and Technology, Xinxiang Vocational and Technical College, Xinxiang 453007, People’s Republic of China

## Abstract

In the title compound, 2[Co(C_2_N_3_)(NO_3_)(C_14_H_12_N_2_)(H_2_O)]·C_14_H_12_N_2_·2H_2_O, the Co^II^ ion is coordinated by a bidentate 2,9-dimethyl-1,10-phenanthroline (dmphen) ligand, a bidentate nitrate anion, a water mol­ecule and a monodentate dicyan­amide group in a distorted octa­hedral geometry. One uncoordinated dmphen mol­ecule is situated on a crystallographic twofold axis and the asymmetric unit is completed by one water mol­ecule. In the crystal, mol­ecules form a one-dimensional framework in the [001] direction through O—H⋯N and O—H⋯O hydrogen bonds. The crystal packing is further stabilized by π–π stacking inter­actions between the dmphen rings of neighboring mol­ecules, with a centroid–centroid separation of 3.5641 (8) Å and a partially overlapped arrangement of parallel dmphen rings with a distance of 3.407 (2) Å.

## Related literature

For background to metal–phenanthroline complexes, see: Naing *et al.* (1995 ▶); Wang *et al.* (1996 ▶); Wall *et al.* (1999 ▶). For related Co(II)–phenanthroline structures, see: Ding *et al.* (2006 ▶); Xuan & Zhao (2007 ▶); Zhao *et al.* (2008 ▶).
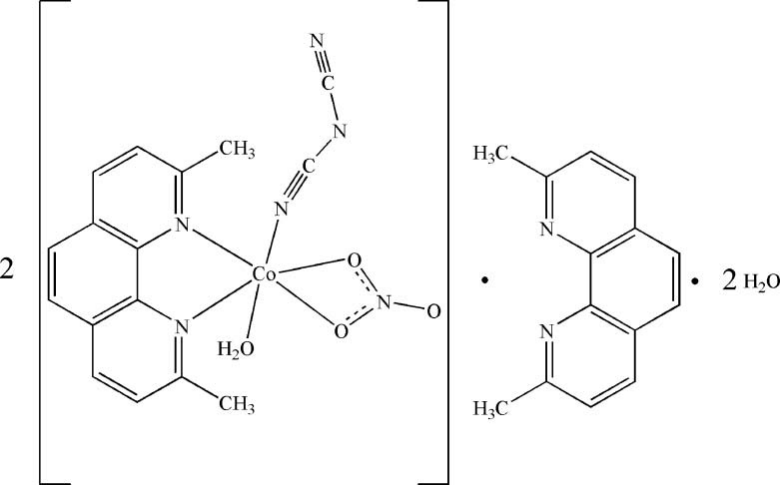


## Experimental

### 

#### Crystal data

2[Co(C_2_N_3_)(NO_3_)(C_14_H_12_N_2_)(H_2_O)]·C_14_H_12_N_2_·2H_2_O
*M* *_r_* = 1070.82
Monoclinic, 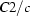

*a* = 17.993 (6) Å
*b* = 11.770 (4) Å
*c* = 23.428 (7) Å
β = 106.981 (4)°
*V* = 4745 (3) Å^3^
*Z* = 4
Mo *K*α radiation
μ = 0.77 mm^−1^
*T* = 291 K
0.34 × 0.18 × 0.11 mm

#### Data collection

Bruker APEXII CCD area-detector diffractometer
Absorption correction: multi-scan (*SADABS*; Bruker, 2004 ▶) *T*_min_ = 0.781, *T*_max_ = 0.917
17592 measured reflections
4400 independent reflections
2862 reflections with *I* > 2σ(*I*)
*R*_int_ = 0.070

#### Refinement

*R*[*F* ^2^ > 2σ(*F* ^2^)] = 0.066
*wR*(*F* ^2^) = 0.196
*S* = 1.04
4400 reflections
328 parameters
36 restraints
H-atom parameters constrained
Δρ_max_ = 1.06 e Å^−3^
Δρ_min_ = −0.58 e Å^−3^

### 

Data collection: *APEX2* (Bruker, 2004 ▶); cell refinement: *SAINT* (Bruker, 2004 ▶); data reduction: *SAINT*; program(s) used to solve structure: *SHELXS97* (Sheldrick, 2008 ▶); program(s) used to refine structure: *SHELXL97* (Sheldrick, 2008 ▶); molecular graphics: *SHELXTL* (Sheldrick, 2008 ▶); software used to prepare material for publication: *publCIF* (Westrip, 2010 ▶).

## Supplementary Material

Crystal structure: contains datablock(s) I, global. DOI: 10.1107/S1600536811035148/bh2369sup1.cif

Structure factors: contains datablock(s) I. DOI: 10.1107/S1600536811035148/bh2369Isup2.hkl

Additional supplementary materials:  crystallographic information; 3D view; checkCIF report

## Figures and Tables

**Table 1:** Hydrogen-bond geometry (Å, °)

*D*—H⋯*A*	*D*—H	H⋯*A*	*D*⋯*A*	*D*—H⋯*A*
O4—H1*W*⋯O5^i^	0.85	2.57	3.111 (9)	123
O4—H2*W*⋯N7	0.85	1.97	2.810 (6)	167
O5—H4*W*⋯N5^ii^	0.85	2.25	2.830 (15)	126

Symmetry codes: (i) 
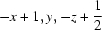
; (ii) 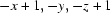.

**Fig. 1.**
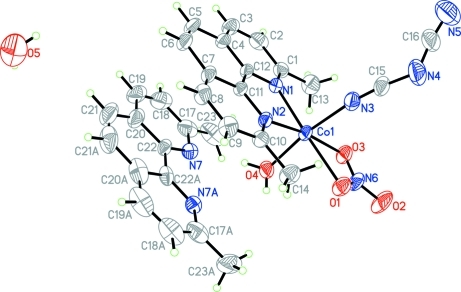
The molecular structure of the title compound, with 30% probability displacement ellipsoids. [Symmetry code: (A) -x + 1, y, -z + 1/2].

**Fig. 2.**
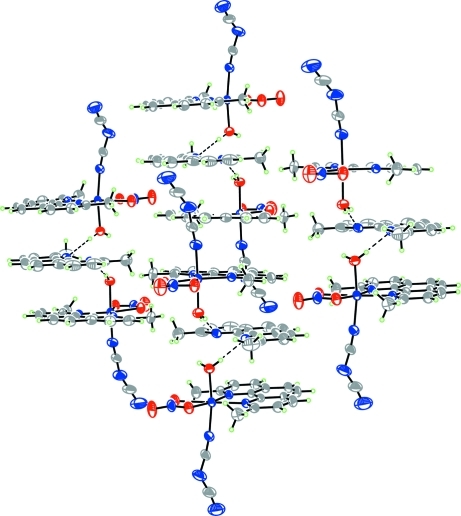
The hydrogen-bonding motifs in the crystal structure. Dashed lines indicate the hydrogen bonds.

**Fig. 3.**
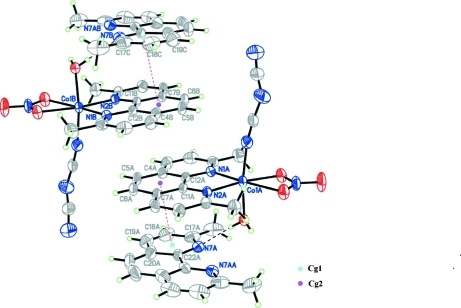
The π-π interaction between the dmphen rings of neighboring molecules in the crystal structure [Symmetry codes: (A) -x + 1, y, -z + 1/2; (B) -x + 1, -y + 1, -z + 1; (C) x, -y + 1, z + 1/2].

## supplementary crystallographic information

### Comment

Metal-phenanthroline complexes and their derivatives have attracted much attention because of their peculiar features (Wang *et al.*, 1996; Wall *et al.*, 1999; Naing *et al.*, 1995). Some Co(II)-phenanthroline complexes have been synthesized and structures were determined (Ding *et al.*, 2006; Xuan & Zhao, 2007; Zhao *et al.*, 2008). Recently, we obtained the title cobalt(II) complex, by reacting 2,9-dimethyl-1,10-phenanthroline, NaN(CN)_2_ and Co(NO_3_)_2_ in an ethanol/water mixture. The structure of the title compound, 2[Co(C_14_H_12_N_2_)N(CN)_2_NO_3_H_2_O]^.^C_14_H_12_N_2_^.^2H_2_O is described below.

Each Co^II^ ion is six-coordinated by two N atoms from a 2,9-dimethyl-1,10-phenanthroline ligand, two O atoms from a nitrate anion, one O atom from a water molecule and one N from a dicyanamide anion. The water and N3/C15/N4/C16/N5 ligands occupy the axial positions, with a N3—Co1—O4 bond angle of 171.71 (16)°. The Co^II^ ion locates in the center, and CoO_3_N_3_ unit forms a distorted octahedral geometry. The uncoordinated dmphen molecule is placed on a crystallographic twofold axis (Fig. 1).

In the crystal structure, molecules are linked into a one-dimensional framework by O—H···N and O—H···O hydrogen bonds (Fig. 2). A partially overlapped arrangement of neighboring parallel Co1A-dmphen and Co1B-dmphen [symmetry code: (A) -*x* + 1, *y*, -*z* + 1/2; (B) -*x* + 1, -*y* + 1, -*z* + 1] rings with distance of 3.4065 (20) Å is observed. The shorter face-to-face separation clearly indicates the existence of π-π stacking between the dmphen rings. Uncoordinated N7A-dmphen and N7B-dmphen rings are also parallel with distance of 10.4286 (19) Å. In addition, the distance between the ring centroids *Cg* (C4A···C7A/C11A/C12A) and *Cg* (C17A···C20A/C22A/N7A) is 3.5641 (8) Å (Fig. 3). This value is close to the van der Waals thickness of the π-π stacking between nearly parallel dmphen (N7A-dmphen with Co1A-dmphen and Co1B-dmphen with N7B-dmphen) rings [dihedral angle: 3.4 (1)°].

### Experimental

NaN(CN)_2_ (0.0892 g, 1 mmol) was dissolved in distilled water (10 ml) and Co(NO_3_)_2_.6H_2_O (0.1456 g, 0.5 mmol) was added. This solution was added to a solution of 2,9-dimethyl-1,10-phenanthroline hemihydrate (C_14_H_12_N_2_.0.5H_2_O, 0.1088 g, 0.5 mmol) in ethanol (10 ml). The mixture was stirred at 323 K and then refluxed for 5 h, cooled to room temperature and filtered. Pink single crystals appeared over a period of 8 days by slow evaporation at room temperature.

### Refinement

Methyl H atoms were placed in calculated positions, with C—H = 0.96 Å, and refined with free torsion angles to fit the electron density; *U*_iso_(H) = 1.5*U*_eq_(carrier C). The water H atoms were located in a differemce map and refined in the riding approximation in their as-found positions and *U*_iso_(H) = 1.5*U*_eq_(carrier O). Other H atoms were placed in calculated positions, with C—H=0.93 Å, and refined in the riding-model approximation with *U*_iso_(H) = 1.2*U*_eq_(carrier C). The final refinement was carried out with 36 restraints on anisotropic displacement parameters for atoms N4, C16, N5, C17, C18, C19, C20 and C21, in order to approximate an isotropic behaviour.

### Crystal data

**Table:**

2[Co(C_2_N_3_)(NO_3_)(C_14_H_12_N_2_)(H_2_O)]·C_14_H_12_N_2_·2H_2_O	*F*(000) = 2208
*M**_r_* = 1070.82	*D*_x_ = 1.499 Mg m^−^^3^
Monoclinic, *C*2/*c*	Mo *K*α radiation, λ = 0.71073 Å
Hall symbol: -C 2yc	Cell parameters from 2628 reflections
*a* = 17.993 (6) Å	θ = 2.4–21.2°
*b* = 11.770 (4) Å	µ = 0.77 mm^−^^1^
*c* = 23.428 (7) Å	*T* = 291 K
β = 106.981 (4)°	Block, pink
*V* = 4745 (3) Å^3^	0.34 × 0.18 × 0.11 mm
*Z* = 4	

### Data collection

**Table:**

Bruker APEXII CCD area-detector diffractometer	4400 independent reflections
Radiation source: fine-focus sealed tube	2862 reflections with *I* > 2σ(*I*)
graphite	*R*_int_ = 0.070
φ and ω scans	θ_max_ = 25.5°, θ_min_ = 2.4°
Absorption correction: multi-scan (*SADABS*; Bruker, 2004)	*h* = −21→21
*T*_min_ = 0.781, *T*_max_ = 0.917	*k* = −13→14
17592 measured reflections	*l* = −28→28

### Refinement

**Table:**

Refinement on *F*^2^	Primary atom site location: structure-invariant direct methods
Least-squares matrix: full	Secondary atom site location: difference Fourier map
*R*[*F*^2^ > 2σ(*F*^2^)] = 0.066	Hydrogen site location: inferred from neighbouring sites
*wR*(*F*^2^) = 0.196	H-atom parameters constrained
*S* = 1.04	*w* = 1/[σ^2^(*F*_o_^2^) + (0.1026*P*)^2^ + 4.8562*P*] where *P* = (*F*_o_^2^ + 2*F*_c_^2^)/3
4400 reflections	(Δ/σ)_max_ < 0.001
328 parameters	Δρ_max_ = 1.06 e Å^−^^3^
36 restraints	Δρ_min_ = −0.58 e Å^−^^3^
0 constraints	

### Fractional atomic coordinates and isotropic or equivalent isotropic displacement parameters (Å^2^)

**Table:**

	*x*	*y*	*z*	*U*_iso_*/*U*_eq_	
Co1	0.42687 (4)	0.19148 (5)	0.38145 (3)	0.0453 (3)	
O1	0.3472 (2)	0.0602 (3)	0.32847 (17)	0.0706 (10)	
O2	0.3720 (3)	−0.1178 (3)	0.3477 (2)	0.1070 (17)	
O3	0.4528 (2)	0.0107 (3)	0.39358 (17)	0.0648 (10)	
O4	0.4792 (3)	0.1851 (3)	0.31525 (17)	0.0672 (11)	
H1W	0.4450	0.1747	0.2819	0.101*	
H2W	0.5124	0.2344	0.3124	0.101*	
O5	0.5612 (6)	−0.0066 (7)	0.2802 (4)	0.233 (5)	
H3W	0.5731	0.0572	0.2975	0.350*	
H4W	0.5602	−0.0359	0.3132	0.350*	
N1	0.5202 (2)	0.2867 (3)	0.43700 (16)	0.0443 (9)	
N2	0.3892 (2)	0.3599 (3)	0.35623 (16)	0.0434 (9)	
N3	0.3653 (3)	0.1776 (4)	0.4424 (2)	0.0694 (13)	
N4	0.2925 (4)	0.1214 (6)	0.5087 (3)	0.114 (2)	
N5	0.3362 (7)	0.1052 (7)	0.6164 (4)	0.172 (4)	
N6	0.3906 (3)	−0.0181 (4)	0.3558 (2)	0.0667 (12)	
N7	0.5696 (2)	0.3653 (4)	0.29332 (18)	0.0534 (10)	
C1	0.5844 (3)	0.2489 (5)	0.4772 (2)	0.0568 (13)	
C2	0.6423 (3)	0.3248 (7)	0.5087 (3)	0.0753 (18)	
H2	0.6866	0.2966	0.5363	0.090*	
C3	0.6345 (3)	0.4380 (6)	0.4995 (3)	0.0701 (16)	
H3	0.6737	0.4871	0.5200	0.084*	
C4	0.5662 (3)	0.4812 (5)	0.4582 (2)	0.0582 (14)	
C5	0.5522 (4)	0.5994 (5)	0.4464 (3)	0.0696 (16)	
H5	0.5887	0.6522	0.4669	0.084*	
C6	0.4872 (4)	0.6351 (5)	0.4062 (3)	0.0694 (16)	
H6	0.4793	0.7125	0.3991	0.083*	
C7	0.4299 (3)	0.5567 (4)	0.3738 (2)	0.0534 (13)	
C8	0.3608 (4)	0.5883 (4)	0.3314 (3)	0.0639 (15)	
H8	0.3504	0.6647	0.3226	0.077*	
C9	0.3089 (3)	0.5090 (5)	0.3029 (3)	0.0651 (15)	
H9	0.2633	0.5308	0.2745	0.078*	
C10	0.3242 (3)	0.3931 (4)	0.3164 (2)	0.0504 (12)	
C11	0.4419 (3)	0.4398 (4)	0.38500 (19)	0.0433 (11)	
C12	0.5111 (3)	0.4015 (4)	0.4278 (2)	0.0455 (11)	
C13	0.5937 (4)	0.1235 (5)	0.4881 (3)	0.0800 (18)	
H13A	0.5582	0.0983	0.5091	0.120*	
H13B	0.6460	0.1073	0.5115	0.120*	
H13C	0.5827	0.0844	0.4505	0.120*	
C14	0.2674 (3)	0.3042 (5)	0.2860 (3)	0.0705 (16)	
H14A	0.2920	0.2521	0.2658	0.106*	
H14B	0.2240	0.3394	0.2576	0.106*	
H14C	0.2495	0.2638	0.3151	0.106*	
C15	0.3341 (4)	0.1530 (5)	0.4758 (3)	0.0691 (16)	
C16	0.3184 (6)	0.1179 (7)	0.5646 (5)	0.110 (3)	
C17	0.6397 (4)	0.3631 (7)	0.3335 (3)	0.0808 (18)	
C18	0.6784 (4)	0.4641 (8)	0.3554 (3)	0.099 (2)	
H18	0.7274	0.4612	0.3830	0.118*	
C19	0.6457 (5)	0.5651 (8)	0.3371 (3)	0.101 (2)	
H19	0.6712	0.6315	0.3534	0.121*	
C20	0.5729 (5)	0.5711 (6)	0.2933 (3)	0.0904 (19)	
C21	0.5337 (5)	0.6744 (6)	0.2707 (4)	0.105 (3)	
H21	0.5565	0.7433	0.2856	0.127*	
C22	0.5374 (3)	0.4659 (4)	0.2729 (2)	0.0542 (13)	
C23	0.6749 (4)	0.2515 (8)	0.3539 (3)	0.111 (3)	
H23A	0.6522	0.1949	0.3245	0.167*	
H23B	0.6655	0.2320	0.3910	0.167*	
H23C	0.7299	0.2550	0.3595	0.167*	

### Atomic displacement parameters (Å^2^)

**Table:**

	*U*^11^	*U*^22^	*U*^33^	*U*^12^	*U*^13^	*U*^23^
Co1	0.0533 (5)	0.0302 (4)	0.0530 (4)	−0.0039 (3)	0.0165 (3)	−0.0007 (3)
O1	0.075 (3)	0.048 (2)	0.079 (3)	−0.002 (2)	0.007 (2)	0.0021 (19)
O2	0.133 (4)	0.035 (2)	0.144 (4)	−0.020 (2)	0.026 (3)	−0.015 (2)
O3	0.070 (3)	0.043 (2)	0.078 (2)	−0.0033 (18)	0.017 (2)	0.0042 (17)
O4	0.104 (3)	0.041 (2)	0.071 (2)	−0.0056 (19)	0.048 (2)	−0.0030 (16)
O5	0.371 (15)	0.145 (7)	0.204 (9)	−0.066 (8)	0.118 (9)	−0.001 (6)
N1	0.047 (2)	0.040 (2)	0.047 (2)	−0.0035 (17)	0.0147 (18)	0.0012 (16)
N2	0.047 (2)	0.035 (2)	0.051 (2)	−0.0026 (18)	0.0189 (19)	−0.0007 (17)
N3	0.078 (3)	0.060 (3)	0.082 (3)	−0.016 (2)	0.041 (3)	−0.010 (2)
N4	0.129 (5)	0.110 (5)	0.136 (5)	−0.022 (4)	0.092 (5)	−0.001 (4)
N5	0.295 (11)	0.124 (7)	0.147 (7)	−0.022 (7)	0.141 (8)	−0.015 (5)
N6	0.084 (4)	0.032 (3)	0.082 (3)	−0.010 (2)	0.022 (3)	−0.004 (2)
N7	0.043 (2)	0.062 (3)	0.058 (2)	−0.001 (2)	0.020 (2)	−0.004 (2)
C1	0.054 (3)	0.064 (3)	0.051 (3)	−0.004 (3)	0.014 (2)	0.001 (3)
C2	0.049 (3)	0.112 (6)	0.059 (3)	−0.008 (3)	0.007 (3)	−0.003 (3)
C3	0.055 (3)	0.078 (4)	0.075 (4)	−0.022 (3)	0.017 (3)	−0.017 (3)
C4	0.060 (3)	0.057 (3)	0.064 (3)	−0.023 (3)	0.028 (3)	−0.017 (3)
C5	0.082 (4)	0.050 (3)	0.083 (4)	−0.032 (3)	0.034 (4)	−0.026 (3)
C6	0.097 (5)	0.037 (3)	0.087 (4)	−0.015 (3)	0.048 (4)	−0.013 (3)
C7	0.071 (4)	0.030 (2)	0.069 (3)	−0.001 (2)	0.036 (3)	−0.006 (2)
C8	0.085 (4)	0.034 (3)	0.081 (4)	0.015 (3)	0.037 (3)	0.009 (3)
C9	0.066 (4)	0.054 (3)	0.076 (4)	0.016 (3)	0.021 (3)	0.008 (3)
C10	0.057 (3)	0.042 (3)	0.053 (3)	0.006 (2)	0.017 (2)	0.002 (2)
C11	0.052 (3)	0.034 (2)	0.050 (3)	−0.005 (2)	0.023 (2)	−0.0062 (19)
C12	0.050 (3)	0.041 (3)	0.052 (3)	−0.009 (2)	0.025 (2)	−0.007 (2)
C13	0.078 (4)	0.073 (4)	0.078 (4)	0.012 (3)	0.007 (3)	0.021 (3)
C14	0.061 (4)	0.066 (4)	0.074 (4)	−0.002 (3)	0.003 (3)	−0.001 (3)
C15	0.079 (4)	0.045 (3)	0.098 (4)	−0.009 (3)	0.049 (4)	−0.010 (3)
C16	0.160 (6)	0.081 (5)	0.128 (6)	−0.018 (4)	0.105 (6)	−0.018 (5)
C17	0.054 (4)	0.130 (6)	0.065 (3)	−0.010 (4)	0.028 (3)	−0.011 (4)
C18	0.066 (4)	0.145 (6)	0.088 (4)	−0.038 (4)	0.028 (3)	−0.032 (4)
C19	0.096 (5)	0.118 (5)	0.106 (5)	−0.066 (4)	0.057 (4)	−0.047 (4)
C20	0.114 (5)	0.072 (4)	0.112 (5)	−0.040 (4)	0.075 (4)	−0.027 (3)
C21	0.152 (7)	0.053 (3)	0.139 (7)	−0.025 (4)	0.085 (5)	−0.023 (4)
C22	0.061 (3)	0.046 (3)	0.071 (3)	−0.014 (2)	0.043 (3)	−0.012 (2)
C23	0.073 (5)	0.174 (8)	0.084 (5)	0.046 (5)	0.019 (4)	0.030 (5)

### Geometric parameters (Å, °)

**Table:**

Co1—O4	2.037 (4)	C5—H5	0.9300
Co1—N3	2.053 (5)	C6—C7	1.425 (8)
Co1—N1	2.120 (4)	C6—H6	0.9300
Co1—N2	2.122 (4)	C7—C8	1.397 (7)
Co1—O3	2.180 (4)	C7—C11	1.405 (7)
Co1—O1	2.224 (4)	C8—C9	1.350 (8)
O1—N6	1.256 (6)	C8—H8	0.9300
O2—N6	1.220 (5)	C9—C10	1.408 (7)
O3—N6	1.253 (6)	C9—H9	0.9300
O4—H1W	0.8501	C10—C14	1.491 (7)
O4—H2W	0.8500	C11—C12	1.425 (7)
O5—H3W	0.8500	C13—H13A	0.9600
O5—H4W	0.8501	C13—H13B	0.9600
N1—C1	1.336 (6)	C13—H13C	0.9600
N1—C12	1.371 (6)	C14—H14A	0.9600
N2—C10	1.325 (6)	C14—H14B	0.9600
N2—C11	1.366 (6)	C14—H14C	0.9600
N3—C15	1.126 (7)	C17—C18	1.398 (10)
N4—C16	1.256 (11)	C17—C23	1.476 (11)
N4—C15	1.276 (8)	C18—C19	1.339 (11)
N5—C16	1.172 (11)	C18—H18	0.9300
N7—C17	1.336 (7)	C19—C20	1.410 (11)
N7—C22	1.344 (7)	C19—H19	0.9300
C1—C2	1.406 (8)	C20—C22	1.411 (8)
C1—C13	1.499 (8)	C20—C21	1.427 (10)
C2—C3	1.349 (9)	C21—C21^i^	1.314 (17)
C2—H2	0.9300	C21—H21	0.9300
C3—C4	1.419 (8)	C22—C22^i^	1.455 (11)
C3—H3	0.9300	C23—H23A	0.9600
C4—C12	1.398 (7)	C23—H23B	0.9600
C4—C5	1.426 (8)	C23—H23C	0.9600
C5—C6	1.337 (9)		
			
O4—Co1—N3	171.71 (16)	C9—C8—C7	120.8 (5)
O4—Co1—N1	91.57 (16)	C9—C8—H8	119.6
N3—Co1—N1	96.13 (17)	C7—C8—H8	119.6
O4—Co1—N2	90.19 (14)	C8—C9—C10	119.8 (5)
N3—Co1—N2	94.27 (17)	C8—C9—H9	120.1
N1—Co1—N2	78.95 (14)	C10—C9—H9	120.1
O4—Co1—O3	86.23 (14)	N2—C10—C9	121.2 (5)
N3—Co1—O3	88.30 (17)	N2—C10—C14	118.1 (4)
N1—Co1—O3	109.58 (15)	C9—C10—C14	120.7 (5)
N2—Co1—O3	170.81 (15)	N2—C11—C7	122.4 (4)
O4—Co1—O1	85.13 (16)	N2—C11—C12	117.9 (4)
N3—Co1—O1	86.69 (17)	C7—C11—C12	119.7 (4)
N1—Co1—O1	167.45 (15)	N1—C12—C4	123.1 (5)
N2—Co1—O1	113.10 (14)	N1—C12—C11	117.5 (4)
O3—Co1—O1	58.18 (14)	C4—C12—C11	119.3 (5)
N6—O1—Co1	91.2 (3)	C1—C13—H13A	109.5
N6—O3—Co1	93.4 (3)	C1—C13—H13B	109.5
Co1—O4—H1W	109.6	H13A—C13—H13B	109.5
Co1—O4—H2W	121.9	C1—C13—H13C	109.5
H1W—O4—H2W	111.2	H13A—C13—H13C	109.5
H3W—O5—H4W	89.8	H13B—C13—H13C	109.5
C1—N1—C12	118.6 (4)	C10—C14—H14A	109.5
C1—N1—Co1	128.6 (3)	C10—C14—H14B	109.5
C12—N1—Co1	112.8 (3)	H14A—C14—H14B	109.5
C10—N2—C11	119.2 (4)	C10—C14—H14C	109.5
C10—N2—Co1	128.1 (3)	H14A—C14—H14C	109.5
C11—N2—Co1	112.8 (3)	H14B—C14—H14C	109.5
C15—N3—Co1	169.5 (5)	N3—C15—N4	173.6 (8)
C16—N4—C15	122.4 (8)	N5—C16—N4	172.3 (10)
O2—N6—O3	121.2 (5)	N7—C17—C18	120.5 (7)
O2—N6—O1	121.6 (5)	N7—C17—C23	118.3 (7)
O3—N6—O1	117.1 (4)	C18—C17—C23	121.2 (7)
C17—N7—C22	119.3 (5)	C19—C18—C17	120.9 (7)
N1—C1—C2	120.9 (5)	C19—C18—H18	119.6
N1—C1—C13	118.5 (5)	C17—C18—H18	119.6
C2—C1—C13	120.6 (5)	C18—C19—C20	120.3 (7)
C3—C2—C1	121.1 (6)	C18—C19—H19	119.8
C3—C2—H2	119.4	C20—C19—H19	119.8
C1—C2—H2	119.4	C19—C20—C22	115.8 (7)
C2—C3—C4	119.4 (5)	C19—C20—C21	124.5 (7)
C2—C3—H3	120.3	C22—C20—C21	119.7 (7)
C4—C3—H3	120.3	C21^i^—C21—C20	121.6 (4)
C12—C4—C3	116.8 (5)	C21^i^—C21—H21	119.2
C12—C4—C5	119.8 (5)	C20—C21—H21	119.2
C3—C4—C5	123.4 (5)	N7—C22—C20	123.2 (6)
C6—C5—C4	120.8 (5)	N7—C22—C22^i^	118.2 (3)
C6—C5—H5	119.6	C20—C22—C22^i^	118.7 (4)
C4—C5—H5	119.6	C17—C23—H23A	109.5
C5—C6—C7	121.3 (5)	C17—C23—H23B	109.5
C5—C6—H6	119.4	H23A—C23—H23B	109.5
C7—C6—H6	119.4	C17—C23—H23C	109.5
C8—C7—C11	116.7 (5)	H23A—C23—H23C	109.5
C8—C7—C6	124.2 (5)	H23B—C23—H23C	109.5
C11—C7—C6	119.1 (5)		
			
O4—Co1—O1—N6	90.4 (3)	C5—C6—C7—C8	179.8 (5)
N3—Co1—O1—N6	−88.2 (3)	C5—C6—C7—C11	0.5 (8)
N1—Co1—O1—N6	15.2 (8)	C11—C7—C8—C9	−0.1 (8)
N2—Co1—O1—N6	178.5 (3)	C6—C7—C8—C9	−179.5 (5)
O3—Co1—O1—N6	1.8 (3)	C7—C8—C9—C10	0.5 (8)
O4—Co1—O3—N6	−88.4 (3)	C11—N2—C10—C9	0.6 (7)
N3—Co1—O3—N6	85.3 (3)	Co1—N2—C10—C9	−177.4 (4)
N1—Co1—O3—N6	−178.8 (3)	C11—N2—C10—C14	−179.6 (4)
O1—Co1—O3—N6	−1.8 (3)	Co1—N2—C10—C14	2.4 (7)
O4—Co1—N1—C1	−90.8 (4)	C8—C9—C10—N2	−0.8 (8)
N3—Co1—N1—C1	86.1 (4)	C8—C9—C10—C14	179.3 (5)
N2—Co1—N1—C1	179.3 (4)	C10—N2—C11—C7	−0.1 (6)
O3—Co1—N1—C1	−4.3 (4)	Co1—N2—C11—C7	178.2 (3)
O1—Co1—N1—C1	−16.3 (9)	C10—N2—C11—C12	179.8 (4)
O4—Co1—N1—C12	87.9 (3)	Co1—N2—C11—C12	−1.9 (5)
N3—Co1—N1—C12	−95.2 (3)	C8—C7—C11—N2	−0.1 (7)
N2—Co1—N1—C12	−2.0 (3)	C6—C7—C11—N2	179.3 (4)
O3—Co1—N1—C12	174.4 (3)	C8—C7—C11—C12	180.0 (4)
O1—Co1—N1—C12	162.4 (6)	C6—C7—C11—C12	−0.6 (7)
O4—Co1—N2—C10	88.7 (4)	C1—N1—C12—C4	0.4 (7)
N3—Co1—N2—C10	−84.3 (4)	Co1—N1—C12—C4	−178.4 (4)
N1—Co1—N2—C10	−179.8 (4)	C1—N1—C12—C11	−179.5 (4)
O1—Co1—N2—C10	3.9 (4)	Co1—N1—C12—C11	1.7 (5)
O4—Co1—N2—C11	−89.4 (3)	C3—C4—C12—N1	1.1 (7)
N3—Co1—N2—C11	97.5 (3)	C5—C4—C12—N1	−179.5 (4)
N1—Co1—N2—C11	2.1 (3)	C3—C4—C12—C11	−179.0 (4)
O1—Co1—N2—C11	−174.2 (3)	C5—C4—C12—C11	0.4 (7)
N1—Co1—N3—C15	−113 (3)	N2—C11—C12—N1	0.2 (6)
N2—Co1—N3—C15	167 (3)	C7—C11—C12—N1	−179.9 (4)
O3—Co1—N3—C15	−4(3)	N2—C11—C12—C4	−179.7 (4)
O1—Co1—N3—C15	55 (3)	C7—C11—C12—C4	0.2 (6)
Co1—O3—N6—O2	−174.5 (5)	C22—N7—C17—C18	1.8 (8)
Co1—O3—N6—O1	3.1 (5)	C22—N7—C17—C23	−178.1 (5)
Co1—O1—N6—O2	174.5 (5)	N7—C17—C18—C19	0.8 (10)
Co1—O1—N6—O3	−3.0 (5)	C23—C17—C18—C19	−179.3 (6)
C12—N1—C1—C2	−1.2 (7)	C17—C18—C19—C20	−2.9 (11)
Co1—N1—C1—C2	177.4 (4)	C18—C19—C20—C22	2.4 (10)
C12—N1—C1—C13	178.9 (4)	C18—C19—C20—C21	−179.6 (7)
Co1—N1—C1—C13	−2.5 (7)	C19—C20—C21—C21^i^	179.3 (9)
N1—C1—C2—C3	0.4 (9)	C22—C20—C21—C21^i^	−2.8 (14)
C13—C1—C2—C3	−179.8 (6)	C17—N7—C22—C20	−2.2 (7)
C1—C2—C3—C4	1.2 (9)	C17—N7—C22—C22^i^	177.2 (5)
C2—C3—C4—C12	−1.9 (8)	C19—C20—C22—N7	0.1 (8)
C2—C3—C4—C5	178.8 (5)	C21—C20—C22—N7	−177.9 (6)
C12—C4—C5—C6	−0.5 (8)	C19—C20—C22—C22^i^	−179.3 (6)
C3—C4—C5—C6	178.8 (5)	C21—C20—C22—C22^i^	2.6 (9)
C4—C5—C6—C7	0.1 (9)		

Symmetry codes: (i) −*x*+1, *y*, −*z*+1/2.

### Hydrogen-bond geometry (Å, °)

**Table:**

*D*—H···*A*	*D*—H	H···*A*	*D*···*A*	*D*—H···*A*
O4—H1W···O5^i^	0.85	2.57	3.111 (9)	123.
O4—H2W···N7	0.85	1.97	2.810 (6)	167.
O5—H3W···O4	0.85	2.39	2.941 (11)	123.
O5—H4W···N5^ii^	0.85	2.25	2.830 (15)	126.

Symmetry codes: (i) −*x*+1, *y*, −*z*+1/2; (ii) −*x*+1, −*y*, −*z*+1.
